# High-Quality Semiconductor Nanocrystals through Eutectic Processing

**DOI:** 10.1021/acs.chemmater.6c02191

**Published:** 2026-10-06

**Authors:** Divesh Nazar, Bhanuka Thennakoon, Siddhartha Kalpa Samadhi Thennakoon, Amelia Waters, Kosgoda Somarathne, Keston Walleman, Ryan Liebowitz, Renown Shrestha, Lydia Boeding, Pavel Anzenbacher, Mikhail Zamkov

**Affiliations:** † The Center for Photochemical Sciences, 1888 Bowling Green State University, Bowling Green, Ohio 43403, United States; ‡ Department of Physics, 1888 Bowling Green State University, Bowling Green, Ohio 43403, United States; § Department of Chemistry, Bowling Green State University, Bowling Green, Ohio 43403, United States

## Abstract

The use of eutectic reactions in colloidal semiconductor nanocrystal (NC) synthesis has recently opened a route to materials with exceptional structural and optical quality. Unlike conventional hot-injection and ripening methods, eutectic-like NC-halide mixtures can promote thermodynamically driven shape evolution through transient, halide-induced recrystallization of semiconductor nanoparticles. This mechanism has proven remarkably versatile, producing a broad range of metal chalcogenide NCs, including alloys and core/shell heterostructures with reduced defect densities and improved photostability. Despite these advances, the chemical pathways governing eutectic-like reconstruction in NCs remain poorly understood, leaving the field in an early stage of development. This Perspective examines the evolving picture of NC growth through halide-induced reconstruction, highlights representative examples of shape and line width control, and discusses the emerging theoretical insights into the underlying mechanisms. We further review evolving applications of eutectic processing in NC light-emitting devices, optical gain media, photovoltaics, and scintillators. Finally, we discuss future opportunities for the field, including broader adoption of eutectic conditions as a general synthetic strategy for colloidal nanomaterials, its extension to challenging compositions such as III–V semiconductors, and eutectic processing of NC films to create epitaxially connected nanoparticle assemblies.

## Graphical abstract

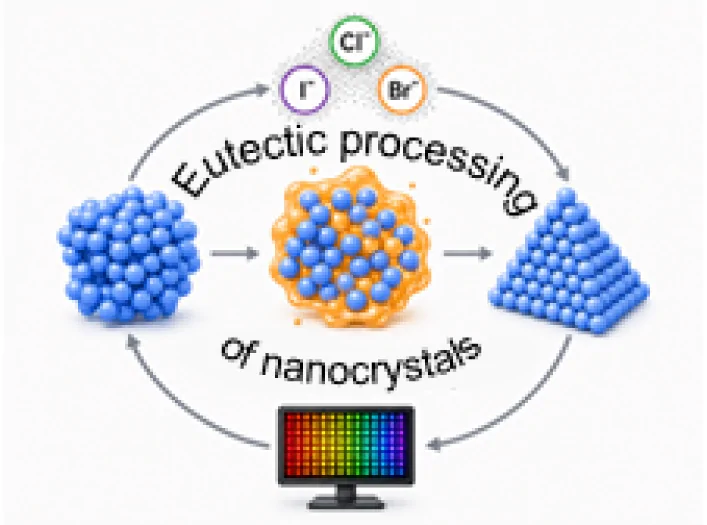

## Introduction

Colloidal semiconductor nanocrystals (NCs) represent a promising class of inorganic nanomaterials for solution processing of optoelectronic devices.^1–9^ A strong correlation between NC geometry and ensuing optoelectronic properties has been the key driving force behind the strong interest in these nanomaterials.^10–12^ Consequently, a substantial synthetic effort has been devoted to controlling the NC size distributions^13–22^ and their structural morphologies.^12,23–25^

Since the first demonstrations of colloidal NCs,^26–28^ most synthetic strategies have relied on a classical paradigm that includes a rapid nucleation of seeds followed by kinetically controlled nanoparticle growth through monomer addition.^29–33^ This framework has been particularly beneficial in materials with more ionic lattice character, such as Cd chalcogenides and inorganic halide perovskites, where less directional electrostatic bonding allows ion reorganization and defect healing under comparatively mild conditions.^34^ Despite its success, such a classical monomer-mediated growth is inherently limited by kinetic trapping, often preventing NCs from reaching their thermodynamic equilibrium. This problem becomes especially significant for semiconductors with stronger covalent bonding character, including group IV and most III–V NCs. Unlike more ionic lattices, covalent semiconductors are stabilized by stronger, more directional bonds, making postgrowth atomic rearrangement energetically demanding. As a result, the final size and shape of these NCs tend to reflect the balance of precursor reactivity and growth rate rather than the true thermodynamic minimum. In many cases, the resulting nanoparticles contain stacking faults and trap-rich interfaces, which limits the ability of conventional hot-injection and ripening methods to produce high-quality NCs. These constraints lead to suboptimal performance in applications requiring high radiative efficiency and photostability, including LEDs,^35^ optical gain media,^36–38^ scintillators,^39,40^ displays,^41^ and quantum light sources.^42,43^

Eutectic growth of NCs provides a direct strategy for overcoming the kinetic limitations of classical synthesis.^44–59^ Instead of relying on monomer addition alone, this approach uses eutectic mixtures of NCs and halide salts to lower the energy barrier for lattice reconstruction (Figure 1a). Under suitable reaction conditions, the NC lattice can enter a transient liquid-like state, driving the removal of surface defects and coercing the particle shape to more stable geometries. This process is different from conventional digestive or Ostwald ripening mechanisms as it involves a chemically mediated transformation in which nanoparticle melting and its thermodynamic reconstruction are driven by phase changes. Related principles have long been used in noncolloidal semiconductor processing, including CdCl_2_-assisted treatment of CdTe solar cells, where the CdCl_2_ annealing step drives grain-boundary fusion^60–63^ producing nearly an order of magnitude enhancement^64^ in polycrystalline CdTe solar cell efficiency. Eutectic chemistry is also central to Au-assisted nanowire growth (e.g., Ge, GaAs) via the vapor–liquid–solid (VLS) mechanism, where Au-semiconductor eutectic mixture dramatically lowers the melting point of the semiconductor.^65^ More recently, eutectic-based approaches have been demonstrated to work during a solution-phase growth, primarily in NCs of Cd, Zn, and Pb chalcogenide semiconductors, where these methods have delivered advanced levels of shape control and defect suppression compared with conventional hot-injection or ripening techniques. These advances have already resulted in high-quality NCs that show excellent performance in X-ray scintillators,^66^ displays,^58^ luminescent solar concentrators,^58^ optical gain media,^59–67^ and other emerging NC technologies.^68^

**Figure 1:**
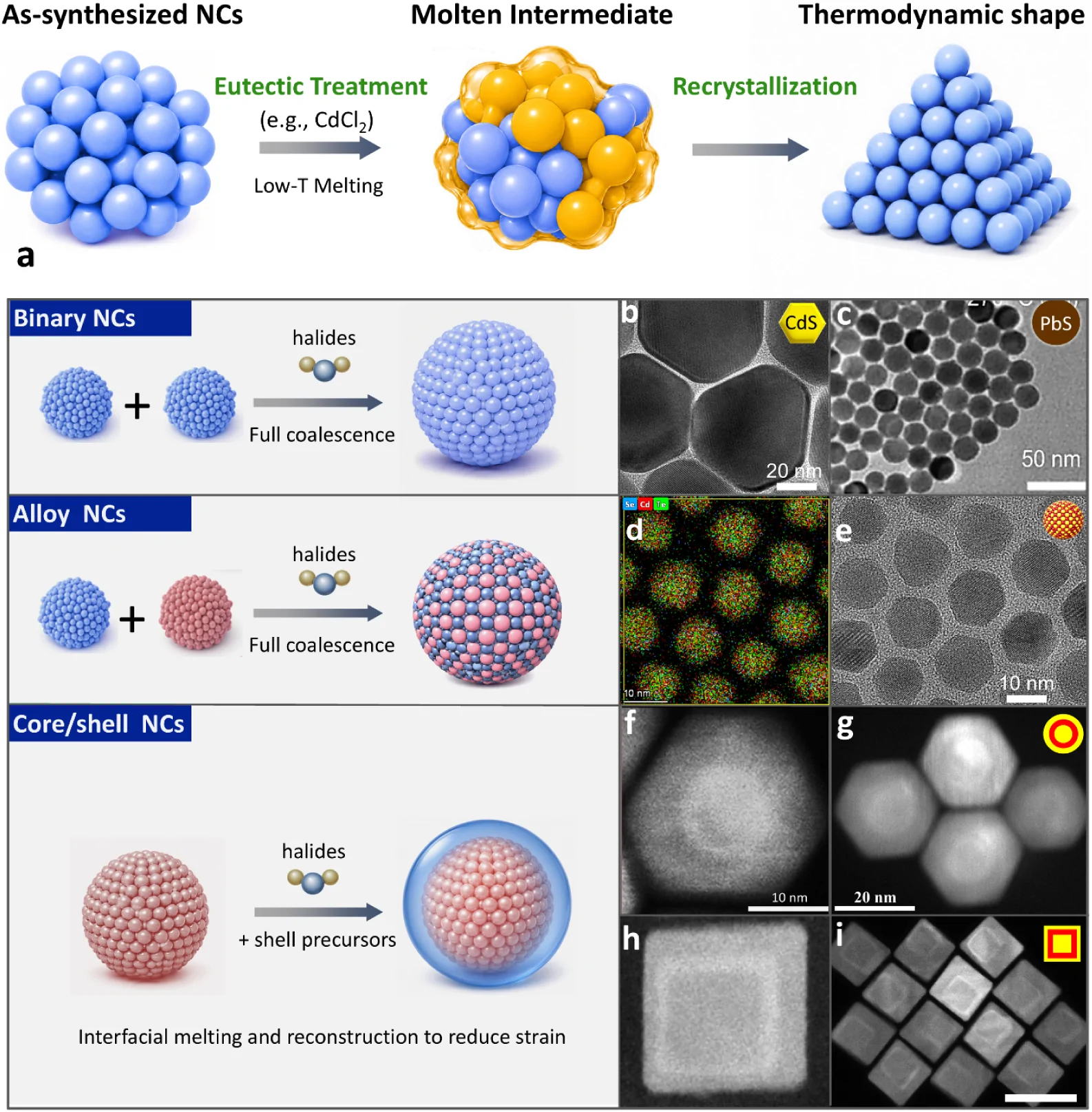
(a) Schematic illustration of eutectic-assisted nanocrystal (NC) reconstruction. The addition of halide salts to a colloidal NC solution forms an NC-halide eutectic system with a reduced melting point, enabling transient liquid-like behavior and subsequent recrystallization into a thermodynamically stable, low-defect crystalline phase. (b-i) TEM images of representative NC architectures accessible through eutectic reconstruction: (b) giant CdS NCs; (c) giant PbS NCs; (d, e) CdSeTe alloy NCs; (f, g) CdS/CdSe/CdS core/shell/shell quantum shells (QSs); and (h, i) cubic CdS/CdSe/CdS core/shell/shell NCs (quantum cubes).

This Perspective examines eutectic reactions in colloidal semiconductor NC synthesis as an emerging route to high-quality materials. We use the term *eutectic* with some caution because, unlike in bulk semiconductors where halides are known to induce a thermodynamic pathway to low-temperature lattice melting, in nanocrystalline form, strong halide coordination may simultaneously modify interfacial phases and reduce kinetic barriers to bond exchanges. In other words, eutectic behavior defines when a highly mobile phase becomes thermodynamically accessible, whereas halide-induced metal-anion bond exchange reactions may determine how rapidly the NC can restructure once that state is reached.

We first contrast eutectic reactions in NCs with classical monomer growth and ripening, providing early examples of this process in solid-state semiconductors. We then outline the fundamental principles of eutectic phase changes in colloidal NCs. Representative examples are considered across binary NCs of II–VI and IV–VI semiconductors, especially larger-diameter nanoparticles for which monodispersity and high lattice quality are difficult to achieve (Figure 1b, c). Next, we consider eutectic processing of ternary alloyed NCs, where transient liquidus state of the lattice promotes homogeneous anion mixing (Figure 1d, e), as well as core/shell NCs, where eutectic softening of the lattice can relieve strain and improve interfacial quality (Figure 1f–i). These demonstrations will be considered in the context of optoelectronic applications, including relevant parallels to the eutectic processing of CdTe solar cells and quantum dot (QD) displays. We will then present a phenomenological description of eutectic reactions in colloidal NCs. The Perspective will conclude by identifying several opportunities for future development, including the adaptation of eutectic chemistry to more covalently bonded III–V semiconductor NCs and the extension of this concept to eutectic processing of epitaxially connected NC films.

### Conventional versus Eutectic-Like NC Growth

The conventional synthesis of colloidal semiconductor NCs relies on controlling the growth through precursor reactivity and the reaction temperature. Other synthetic parameters, including monomer supply, surface chemistry, monomer stability, and so on, also come into play. When these parameters are carefully balanced, monodisperse nanoparticles can be obtained.^13–17^ Yet, the success of nucleation methods often hides an important limitation: many NCs are kinetically selected rather than thermodynamically derived.^12,69^ Under these conditions, particles may adopt metastable shapes with strained interfaces and compositional gradients. Subsequent ripening methods, such as Ostwald ripening^70^ (dissolution of smaller NCs in favor of larger ones) or digestive ripening^71^ (redistribution toward smaller sizes), may improve some features, but these are generally difficult to direct. These constraints reflect a deeper issue that once a covalent or partially covalent lattice forms, conventional reaction conditions often do not provide sufficient atomic mobility to eliminate defects.^72^ Eutectic processing directly targets this missing step by creating a local environment in which partial reconstruction is possible.

A eutectic mixture is a composition of two or more components that melts at a temperature below that of the individual constituents. In bulk semiconductors, eutectic principles have long been exploited to promote crystallization and defect removal under conditions that would otherwise be too mild to reorganize a rigid lattice. For instance, eutectic interactions play a central role in the Au-catalyzed VLS growth of Si and Ge nanowires.^65,73^ During growth, a nanoscale Au catalyst particle absorb semiconductor atoms supplied from the vapor (or liquid) phase and form liquid Au–Si or Au–Ge alloys at temperatures far below the melting points of the pure components. This causes a crystalline semiconductor to precipitate selectively at the liquid–solid interface, making Au–Ge or Au–Si eutectic act as a low-temperature flux that lowers the barrier to crystallization. The VLS mechanism is one of the most representative examples of how eutectic formation can temporarily convert a rigid semiconductor into a mobile liquid-phase intermediate, enabling controlled reconstruction and crystal growth under conditions substantially milder than those required to melt bulk Ge or Si. A related precedent for chemically enhanced lattice mobility was established by Colvin and coworkers in Ti- and Zr-based oxides,^74^ where residual metal halides acted as crystallization catalysts rather than being consumed during synthesis. By functioning as strong Lewis acids, the halides promoted reversible disruption and reformation of M-O bonds, thereby increasing lattice mobility and enabling initially amorphous oxides to reorganize into crystalline phases at reduced temperatures. Another prominent example of semiconductor eutectics is found in CdTe thin-film photovoltaics, where the presence of CdCl_2_ lowers the effective reconstruction temperature of CdTe from the bulk melting point of approximately 1040 °C down to 400 °C, enabling low-temperature lattice reorganization and film sintering.^60–64,75,76^ Such chloride-assisted reorganization has been central to the dramatic improvement in polycrystalline CdTe solar cell performance.

Early demonstrations of halide-assisted NC synthesis came from the pioneering work of Hyeon and coworkers^77^ followed by studies on chloride-treated NC films that provided clear evidence of enhanced lattice mobility in NCs. For instance, Owen and coworkers^78^ reported that chloride-passivated CdSe NC films undergo interparticle fusion and grain growth at unexpectedly low annealing temperatures. Related observations were also made by Talapin, Luther, and coworkers, who showed that CdCl_2_ passivation of CdTe NCs facilitates low-temperature sintering.^79^ At the time, these phenomena were primarily interpreted in terms of chloride surface termination, which promotes ligand removal, causing thermally activated sintering. Their possible connection to nanoscale eutectic softening and reconstruction, however, has not yet been recognized.

A compelling evidence that halides could induce liquid-like behavior in colloidal semiconductors emerged from solution-phase NC coalescence. One study found that CdS­(e) NCs could undergo rapid liquid-like coalescence into larger nanoparticles in oleylamine,^46^ an effect that was later traced to halide impurities rather than pure oleylamine itself. Cassidy and coworkers^45^ subsequently identified CdCl_2_, as a key driver of NC coalescence, although other halides were also found to promote similar transformations. In parallel, Cademartiri and coworkers reported halide-induced coalescence in PbS NCs,^47^ suggesting that this chemistry was not limited just to a group II–VI material family. Similarly, several works have demonstrated a partial coalescence of colloidal NCs in the form of dimers^80,81^ and honeycomb assemblies.^82^ Recent reports have also shown that III–V colloidal NCs can undergo a partial lattice recrystallization when treated by halide salts in solution.^83^

The mechanistic connection between NC lattice transformations in colloidal solutions and the well-established solid-state semiconductor processing became clearer through a recent study involving First Solar, Inc. as an industrial partner.^58^ It was determined that CdCl_2_-enabled eutectic interactions with Cd chalcogenides,^84^ utilized by CdTe photovoltaics, also occur in colloidal NCs, lowering their effective melting point.^58^ Namely, colloidal NCs enter a “liquid-like” eutectic phase, upon mixing with halide salts, which may involve complete melting of the entire NC or its partial wetting (Figure 2). Partial liquification may occur locally at strained regions, including surfaces, interfaces, grain boundaries, high-energy facets, or defect-rich domains. These locations are particularly susceptible to eutectic softening because they contain undercoordinated atoms or nonequilibrium bonding configurations. It was also determined^58^ that a halide component in the eutectic mixture acts as a strong Lewis acid capable of coordinating anion-rich lattice sites, thereby weakening directional covalent bonds and facilitating local bond rearrangement (Figure 2). In this sense, eutectic processing can be viewed as the design of a nanoscale flux that locally converts a rigid, directional covalent lattice into a more ionic and labile coordination environment, lowering kinetic barriers to defect removal, shape relaxation, and structural reorganization.

**Figure 2:**
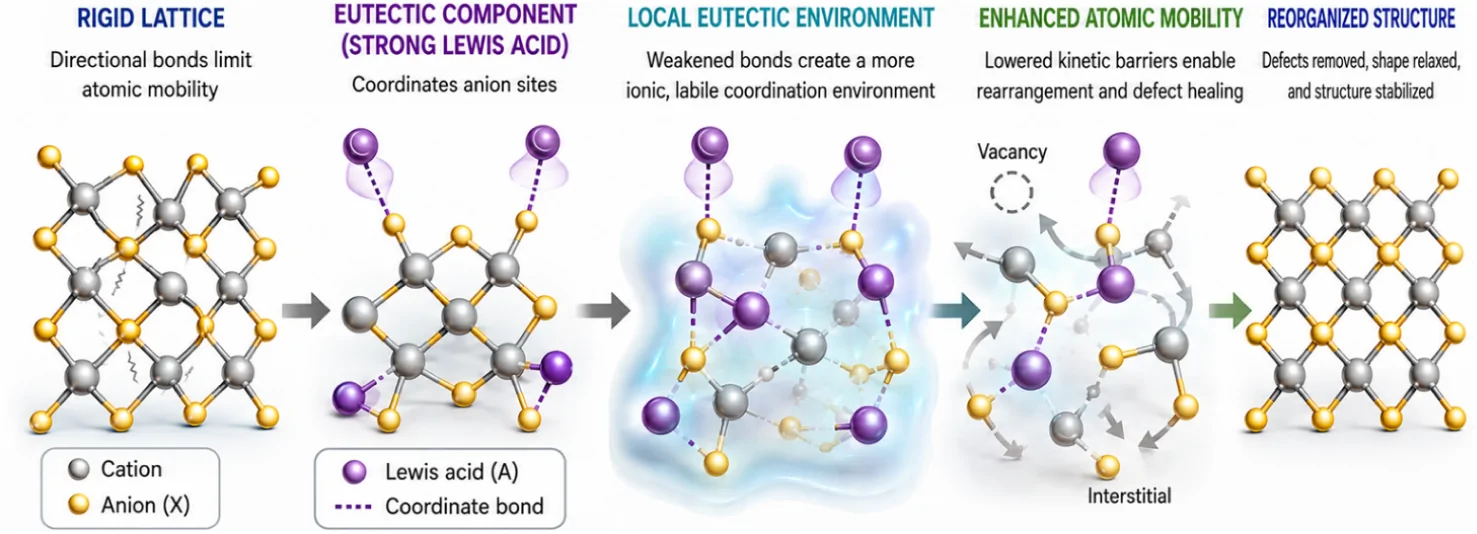
Proposed mechanism of eutectic-assisted lattice reconstruction. A distorted, kinetically trapped semiconductor lattice contains strained bonds and defects with limited atomic mobility. A Lewis-acidic eutectic component weakens directional bonds, creating a more ionic and labile local environment. This transient liquid-like state lowers kinetic barriers to vacancy and interstitial migration. Subsequent recrystallization removes defects, relaxes lattice strain, and produces a more ordered and thermodynamically stable structure. Overall, the eutectic acts as a nanoscale flux that converts a rigid covalent lattice into a dynamically reconfigurable coordination environment, enabling defect healing at reduced temperatures.

### Eutectic Growth of Binary Semiconductor NCs

The clearest evidence for halide-induced lattice softening in colloidal nanoparticles comes from the liquid-like coalescence of binary semiconductor NCs. Lattice softening alone, however, is not sufficient to induce coalescence—nanoparticles must also be driven into sufficiently close contact for fusion to occur. This driving force can be provided by the reaction medium. Coordinating solvents such as oleylamine and oleic acid are mildly polar, whereas the ligand-coated NC surface is largely nonpolar, creating an unfavorable solvent–particle interaction that promotes particle aggregation. Once softened NCs are brought into contact, they can fuse and recrystallize into larger single-crystalline particles. Such a transformation of initially discrete NCs into continuous crystalline domains provides one of the clearest experimental signatures of a transient liquid-like state. Recent studies of Cd-, Pb-, and Zn-chalcogenide NCs have shown that eutectic reactions can promote this type of particle fusion and subsequent recrystallization.^58,59^ These systems also provide an ideal testing ground of eutectic interactions because their optical properties are highly sensitive to the size distribution. The following sections review several representative demonstrations of eutectic-assisted coalescence in these material families.

In a showcase study^58^ demonstrating eutectic-like phase transitions in colloidal NCs, small-diameter CdS NCs were combined with a CdCl_2_ salt in OLAM and gradually heated to approximately 320 °C while monitoring the particle size. TEM images taken at successive reaction stages (Figure 3a) reveal a stepwise growth of CdS NCs from 3.5 nm (30 °C) to 4.8 nm (190 °C), 9.4 nm (260 °C), and 18.4 nm (320 °C). Importantly, no signatures of Ostwald ripening^70^ in which smaller particles dissolve in favor of larger ones, or digestive ripening^71^ in which the ensemble redistributes toward smaller sizes were observed. Instead, after only 3 min at each reaction temperature, the NC population converged to a single, larger particle size, revealing a monocrystalline lattice and with a standard size deviation below 7%. This behavior is consistent with a direct coalescence of liquid-like NCs into a thermodynamically preferred size determined solely by the reaction temperature (Figure 3a).

**Figure 3:**
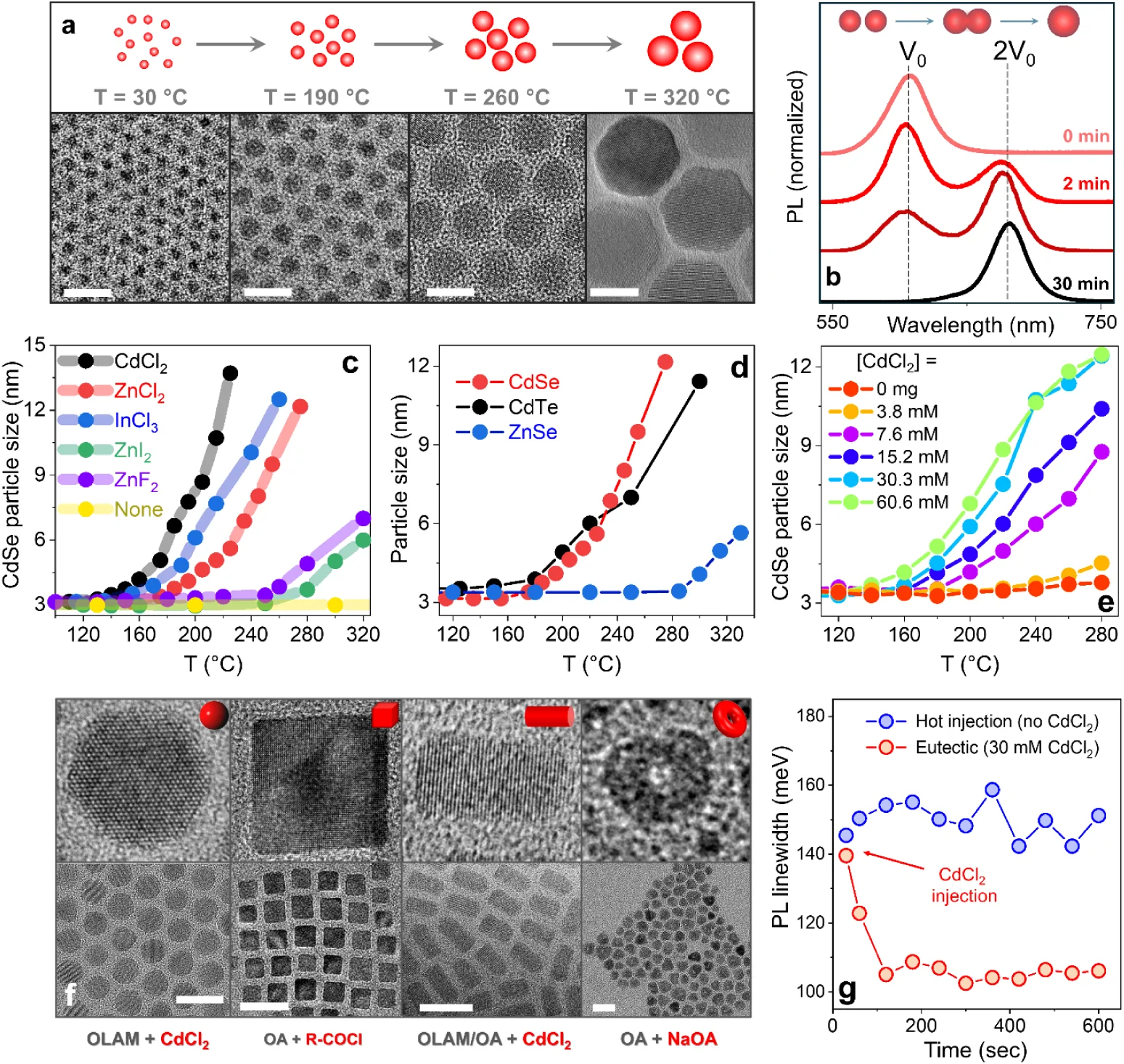
(a) Schematic representation of the eutectic coalescence mechanism, accompanied by representative TEM images of CdS NCs at different temperatures grown in a CdCl_2_/OLAM mixture. Increasing the reaction temperature produces discrete size states, with NC diameters increasing from 3.5 nm to 4.8, 9.4, and 18.4 nm. Scale bars: 10 nm. Each temperature yields a single, uniform particle size corresponding to a thermodynamically preferred state. (b) Coalescence of zinc-blende CdSe NCs near 200 °C, showing a gradual doubling of a nanoparticle volume. (c) Effect of halide type on the temperature-dependent growth of 3 nm CdSe NCs in OLAM. In the absence of halides, no appreciable size evolution is observed, whereas halide additives promote coalescence. CdCl_2_ produces the fastest particle-size increase, while ZnI_2_ and ZnF_2_ lead to slower growth. (d) Size evolution of ∼3 nm CdSe, CdTe, and ZnSe NCs heated in an OLAM/ZnCl_2_ mixture. CdSe and CdTe show similar coalescence onsets near 170 °C, whereas ZnSe requires substantially higher temperatures, with an onset near 280 °C. (e) Dependence of CdSe eutectic growth on CdCl_2_ concentration in OLAM. (f) Shape-selective synthesis of CdS NCs by halide-assisted aggregative growth. Approximately 3 nm zinc-blende CdS seeds were coalesced under different ligand/halide environments, where the coordinating surfactant dictates facet stabilization during recrystallization, yielding four distinct morphologies: spheres obtained in OLAM with CdCl_2_ at 260 °C; cubes produced in OA with a stearoyl chloride additive (C_17_H_35_COCl) at 295 °C; rods grown in OA+ OLAM/CdCl_2_ at 200 °C; doughnut-like (torus) particles synthesized in OA + sodium oleate (NaOA) at 245 °C. All panels: scale bars = 20 nm. Panel (a, b, c, d, e, g) adapted from ref 58 available under a CC BY 4.0 license. Copyright 2026 The Authors. Panel (f) adapted with permission from ref 45. Copyright 2022 American Chemical Society.

Further evidence of liquid-like NC coalescence is provided by slow-growth experiments shown in Figure 3b, performed just above the coalescence threshold temperature, *T*_*th*_. When approximately 3 nm CdSe NCs are heated in the presence of halides to about 200 °C, the original photoluminescence (PL) peak gradually decreases while a new red-shifted feature emerges. The position of this new band is consistent with an approximately 2-fold increase in the particle volume, as expected from the coalescence of two similarly sized NCs. The coexistence of two discrete emissive populations strongly supports a stepwise coalescence mechanism rather than continuous dissolution and reprecipitation. Importantly, no intermediate populations corresponding to fractional multiples of the initial NC volume are observed in Figure 3b, indicating that the particles transform directly into the size state favored at a given temperature. Likewise, the absence of distinct populations associated with the random coalescence of three or four starting particles argues against uncontrolled stochastic aggregation. Collectively, these observations indicate that halide-induced coalescence proceeds through discrete thermodynamic size states, with reaction temperature serving as the primary selector of the final NC dimensions.

The influence of halide type on the coalescence growth is further examined in Figure 3c, which tracks particle size evolution during eutectic synthesis. When ∼3 nm CdSe NCs are heated in pure OLAM, without halide additives, no measurable size change is observed even at the highest temperatures studied (Figure 3c, yellow curve). This confirms that OLAM alone does not induce coalescence. By contrast, all halide-containing reactions show pronounced, temperature-dependent increases in NC size. Among the salts examined, CdCl_2_ exhibits the lowest thermal threshold for coalescence, initiating growth below 200 °C (gray curve), whereas ZnI_2_ and ZnF_2_ require higher temperatures, approximately 250 °C, to achieve comparable particle enlargement. The semiconductor composition also strongly affects the coalescence threshold. As shown in Figure 3d, CdSe and CdTe NCs exhibit nearly identical growth behavior in a ZnCl_2_/OLAM mixture, with coalescence beginning sharply near 170 °C. In contrast, similarly sized ZnSe NCs remain stable to substantially higher temperatures, with growth initiating only near 280 °C. Halide concentration further modulates both the onset and rate of coalescence. As shown in Figure 3e, increasing the CdCl_2_ concentration progressively lowers the threshold temperature, T_th_, until the effect saturates at [CdCl_2_] > 10 mM.

One particularly useful feature of eutectic growth conditions is the ability to control NC shapes. When nanoparticles enter a transient liquid-like phase, their subsequent recrystallization is influenced by the surrounding coordination environment. This is because ligands bind selectively to particular crystallographic facets, lowering the corresponding facet-specific surface free energies.^85^ The magnitude of the ligand-induced surface energy reduction can be expressed as the energy difference between the ligand-bound and unbound surface: *E*_*bound ligand*_ (hkl) – *E*_*free ligand*_.^24^ Consequently, the final shape of an NC is determined by the competition between bulk lattice energy and ligand-modified facet energies, consistent with a Wulff-type construction.^86–88^ Figure 3f illustrates this principle by showing that different surfactants can direct the same semiconductor to recrystallize into distinct, well-faceted structures during eutectic growth.^45^ In particular, OLAM favors the wurtzite phase producing spherical or hexagonally faceted particles, whereas OA promotes formation of the zinc-blende phase with cubic morphologies. By varying the relative concentrations of OA and OLAM, one can also obtain intermediate structures, such as nanorods shown in Figure 3f. We further found that NC coalescence in the presence of NaOA leads to unusual donut-like structures with a central void visible in most imaged NCs. These shapes may be difficult to obtain through conventional kinetically controlled monomer growth.

Eutectic growth conditions can also improve the size uniformity of an NC ensemble. Figure 3g compares CdSe growth with and without CdCl_2_ under otherwise identical conditions. In a conventional hot-injection reaction carried out at 260 °C, the PL line width remains nearly constant throughout the growth process (blue curve), indicating limited size focusing. In contrast, the addition of CdCl_2_ produces an approximately 40% reduction in PL line width (red curve), consistent with a pronounced narrowing of the particle-size distribution. This improvement is directly relevant to energy downconversion applications, including display phosphors^89^ and luminescent solar concentrators,^90^ where narrow emission bands enhance color purity and spectral selectivity. Importantly, the observed line width narrowing occurs without the prolonged ripening times typically required in conventional syntheses, suggesting that eutectic reconstruction bypasses key kinetic barriers to ensemble homogenization.

The synthesis of lead chalcogenide NCs provides another example in which eutectic interactions can overcome the limitations of conventional colloidal growth. PbS and PbSe NCs are now widely investigated for infrared optoelectronics, with synthetic methods yielding highly reproducible NCs featuring narrow size distributions and well-resolved excitonic absorption features.^91,92^ These approaches are particularly effective for particles in the 4–8 nm size range. However, the formation of larger PbS and PbSe NCs is synthetically challenging due to inherent limitations of monomer-mediated growth for these semiconductors. Eutectic synthesis offers a unique route around these limitations by allowing smaller NC seeds to coalesce and reconstruct into larger, highly crystalline particles relevant to near- and shortwave infrared applications.

The early report of PbS NC coalescence growth came from Cademartiri and co-workers^47^ in which they observed that NC fusion can be a significant mechanism of nanoparticle growth in solution (Figure 4a–c). In this approach, oleylammonium hydrosulfide was injected into a PbCl_2_/oleylamine slurry over a range of temperatures, and the resulting products were monitored as a function of the reaction time. Rapid release and consumption of H_2_S produced PbS seeds, while excess sulfur precursor escaped from the reaction mixture, maintaining conditions of a very low supersaturation. This design largely suppresses classical growth through continuous ion or molecular addition to crystal facets, allowing subsequent changes in the particle size and morphology to be attributed primarily to interparticle attachment, fusion, and reconstruction.

**Figure 4:**
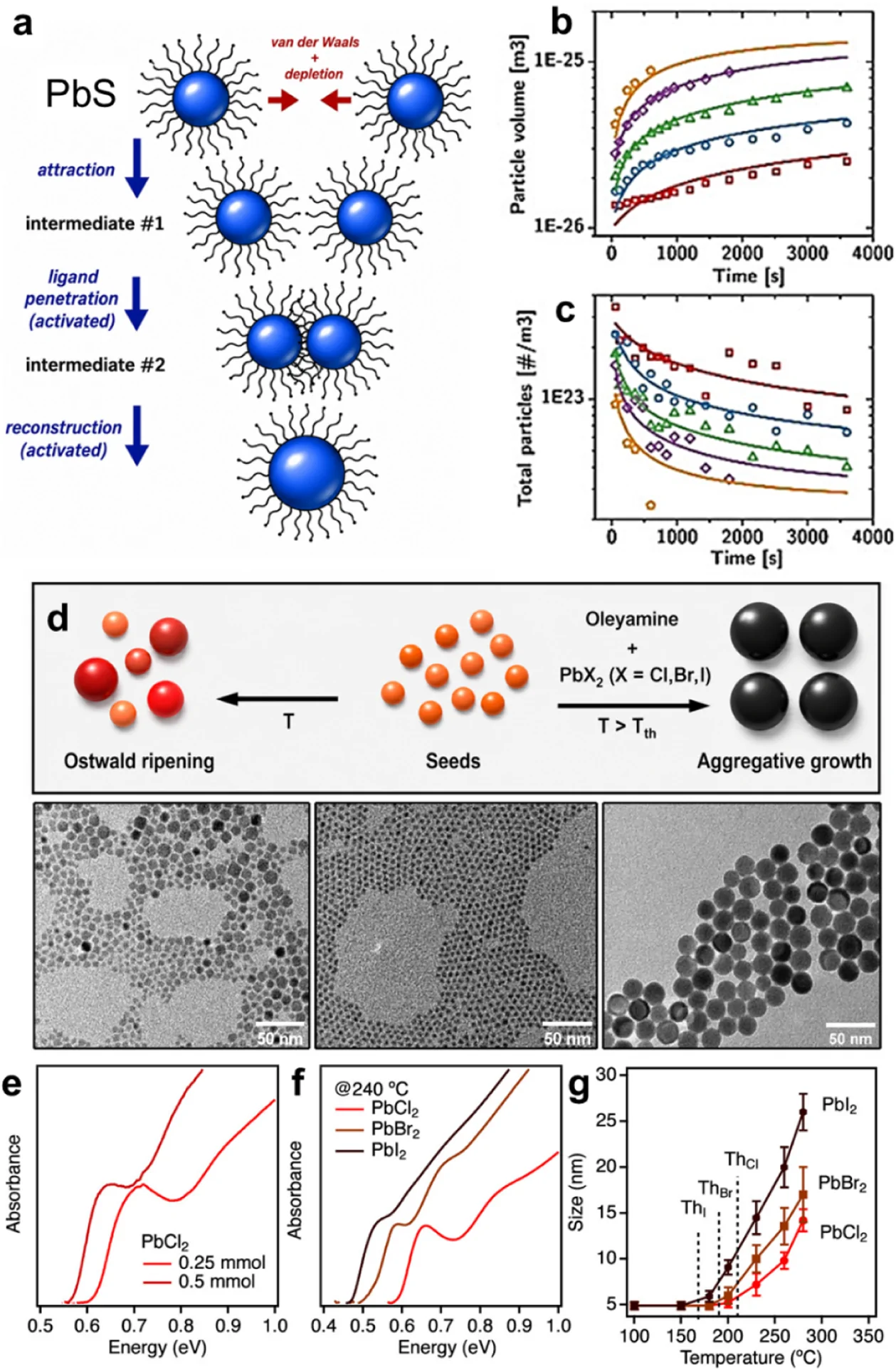
(a) Schematic representation of the two-step NC coalescence mechanism, consisting of interpenetration of the surface ligand shells followed by NC-NC attachment and structural reconstruction. (b, c) Comparison of the experimental kinetic data (open symbols) with model predictions (solid lines) for the number-averaged particle volume (b) and particle concentration (c). Data were collected at 80 °C (red), 100 °C (blue), 120 °C (green), 140 °C (purple), and 160 °C (orange). (d) Representative TEM images of the initial PbS seed NCs (center) and products synthesized in the absence (left) or presence (right) of PbCl_2_. Scale bar: 50 nm. (e) Absorption spectra of PbS NCs synthesized using two different PbCl_2_ amounts, 0.25 and 0.50 mmol, illustrating the effect of halide concentration on particle growth. (f) Absorption spectra of PbS NCs prepared with different lead-halide sources: PbCl_2_, PbBr_2_, and PbI_2_. (g) Dependence of the aggregative-growth threshold temperature on halide identity, denoted (Th_Cl_), (Th_Br_), and (Th_I_) for chloride-, bromide-, and iodide-containing reactions, respectively. Panels (a, b, c) adapted with permission from ref. 47. Copyright 2021 German Chemical Society. Panels (d, e, f, g) reproduced from ref. 59 Copyright 2026 American Chemical Society.

More recently, Geiregat and co-workers^59^ have employed an aggregative growth of PbS NCs to fabricate a new class of solution-processed “bulk infrared nanocrystals” (BINCs). Starting from approximately 4 nm PbS seeds, they developed a lead-halide-assisted (PbX_2_; X = Cl, Br, I) synthesis that yielded highly crystalline, colloidally stable particles as large as 30 nm (Figure 4d, g). Systematic variation of temperature, ligand environment, seed concentration, and halide composition showed that lead halides are essential for initiating aggregative growth and controlling both the final particle size and size distribution. The resulting PbS BINCs exhibited nearly bulk-like infrared properties (Figure 4e, f) without pronounced sub-bandgap trapping, indicating high structural and electronic quality.

### Eutectic Growth of Alloyed NCs

Alloying different semiconductors provides a powerful strategy for tuning the band gaps and lattice constants of NCs. In practice, however, achieving homogeneous alloy formation during the colloidal synthesis remains challenging. This process is often limited by differences in precursor reactivities and diffusion rates, as well as differences in ion sizes. As a result, nominally alloyed NCs may contain compositional gradients.^93–95^ Establishing eutectic conditions during synthesis can overcome these challenges by offering a liquid-like reconstruction of the lattice to drive a more homogeneous phase mixing. In a softened lattice, cations and anions have greater mobility, allowing an NC to approach a more equilibrated alloy composition.

A representative example of employing eutectics to aid alloying was recently demonstrated through the synthesis of CdTe_*x*_Se_1–*x*_ NC colloids. This ternary semiconductor exhibits a strong optical bowing effect, enabling near-infrared emission in the 750–900 nm range.^96,97^ To promote homogeneous Se–Te anion mixing, small-sized CdSe and CdTe NCs were combined in the presence of halide salts (Figure 5a). When heated with either CdCl_2_ or ZnCl_2_, the original NCs coalesced into a single alloy phase, as indicated by the disappearance of distinct CdSe- and CdTe-like features in PL spectra (Figure 5d). This process reproducibly yielded CdTe_*x*_Se_1–*x*_ NCs with emission line widths (∼0.1 eV) narrower than those resulting from optimized hot-injection syntheses (>0.15 eV, see refs. 97–100 ), and PL quantum yields of over 80% for best performing samples. The PL of CdTe_*x*_Se_1–*x*_ NCs could be tuned from 675 to ∼900 nm by varying the initial CdTe:CdSe nanoparticle ratio. TEM analysis of the alloyed NCs revealed well-defined, thermodynamically shaped morphologies (Figure 5c), while STEM-EDAX mapping confirmed homogeneous Te and Se distributions throughout the particles (Figure 5b).

**Figure 5:**
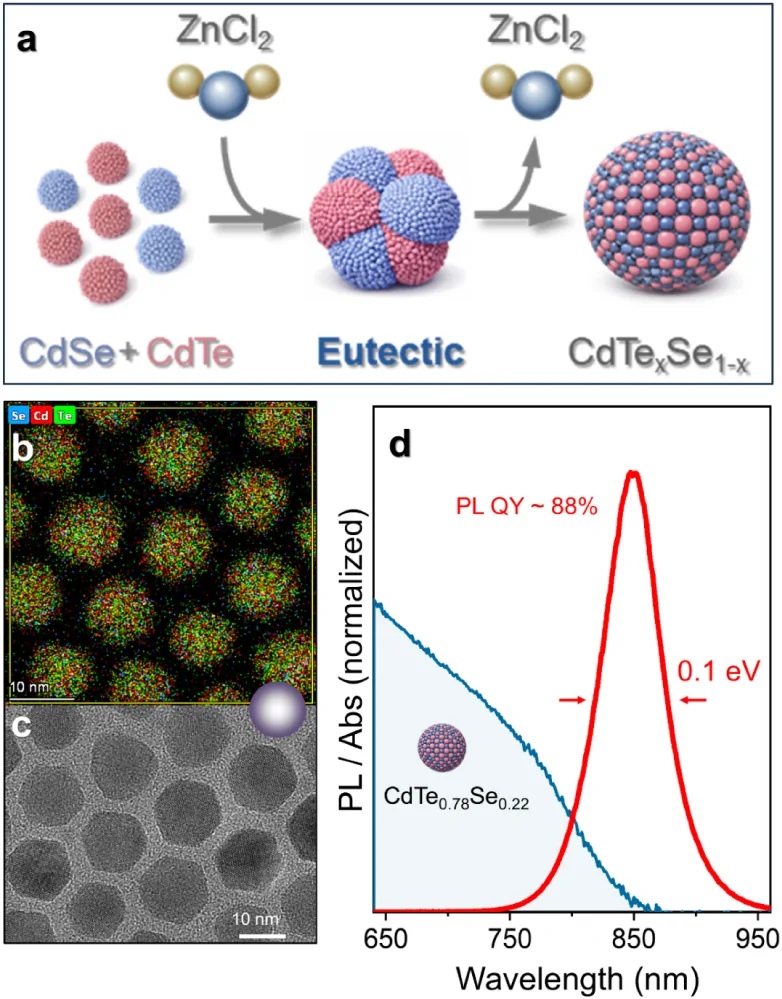
Eutectic-assisted formation of ternary alloy NCs. (a) Schematic illustration of the ternary alloy synthesis, in which presynthesized CdSe and CdTe NCs are heated above the coalescence threshold in a ZnCl_2_/OLAM mixture, leading to fusion and reconstruction into a single CdTe*_x_* Se_1–*x*_ alloy phase. (b) STEM-EDX elemental map showing homogeneous anion distribution across the CdTe_0.78_Se_0.22_ NC alloy. (c) Representative TEM images of CdTe*_x_* Se_1–*x*_ QDs exhibiting thermodynamically faceted morphologies. (d) Absorption and PL spectra of the alloyed CdTe*_x_* Se_1–*x*_ NCs confirming the absence of isolated CdSe and CdTe NCs. Adapted from ref. 58 available under a CC BY 4.0 license. Copyright 2026 The Authors.

### Eutectic Synthesis of Core/Shell Semiconductor NCs

It is well known that the deposition of a semiconductor shell onto colloidal NCs plays an important role in enhancing their optical properties. A shell layer can greatly improve the PL quantum yields or enhance photoinduced charge separation. To date, these advantages have been exemplified through many core/shell architectures, such as CdSe/ZnS,^2^ InP/ZnSe,^9,101^ PbSe/CdSe^102^ and CdTe/CdS NCs.^103^ At the same time, shell growth remains one of the most challenging steps in colloidal NC synthesis. Lattice mismatch between the core and shell materials introduces strain,^104^ while rapid shell deposition can generate interfacial defects and dislocations. Eutectic processing can address these limitations by promoting interfacial reconstruction of strained bonds at the core/shell boundary or by enhancing atomic mobility *during* shell formation, thereby reducing the likelihood of kinetically trapped defects. Below, we provide two recent examples of defect remediation in core–shell NCs using NC eutectics.

Ternary CdTe_*x*_Se_1–*x*_ NCs described above represent a particularly challenging “model system” for shell deposition. Nearly all available wide-bandgap shell semiconductors impose a substantial lattice mismatch. Even the closest matched Zn_*y*_Cd_1–*y*_Se composition for a CdTe_0.78_Se_0.22_ core still exhibits a 5–9% strain depending on the composition variable *y*. As a result, conventional shell growth yields somewhat defective heterostructures with a PL QY of less than 10% (Figure 6a, blue curve). In contrast, introducing a halide salt (ZnCl_2_) into the same reaction medium fundamentally changes the growth pathway. Under properly tuned eutectic conditions, the local melting point at the core/shell interface is reduced without reconstructing the entire NC, which leads to strain-relieved shell deposition and increased PL QY of ∼86% (Figure 6a (red curve) and 6c). The improved shell quality is further evidenced by microscopy images of core/shell NCs grown in the presence of CdCl_2_ (Figure 6d, e) or ZnCl_2_ (Figure 6f–h), which reveal well-faceted, high-symmetry morphologies characteristic of thermodynamically evolved shells.

**Figure 6:**
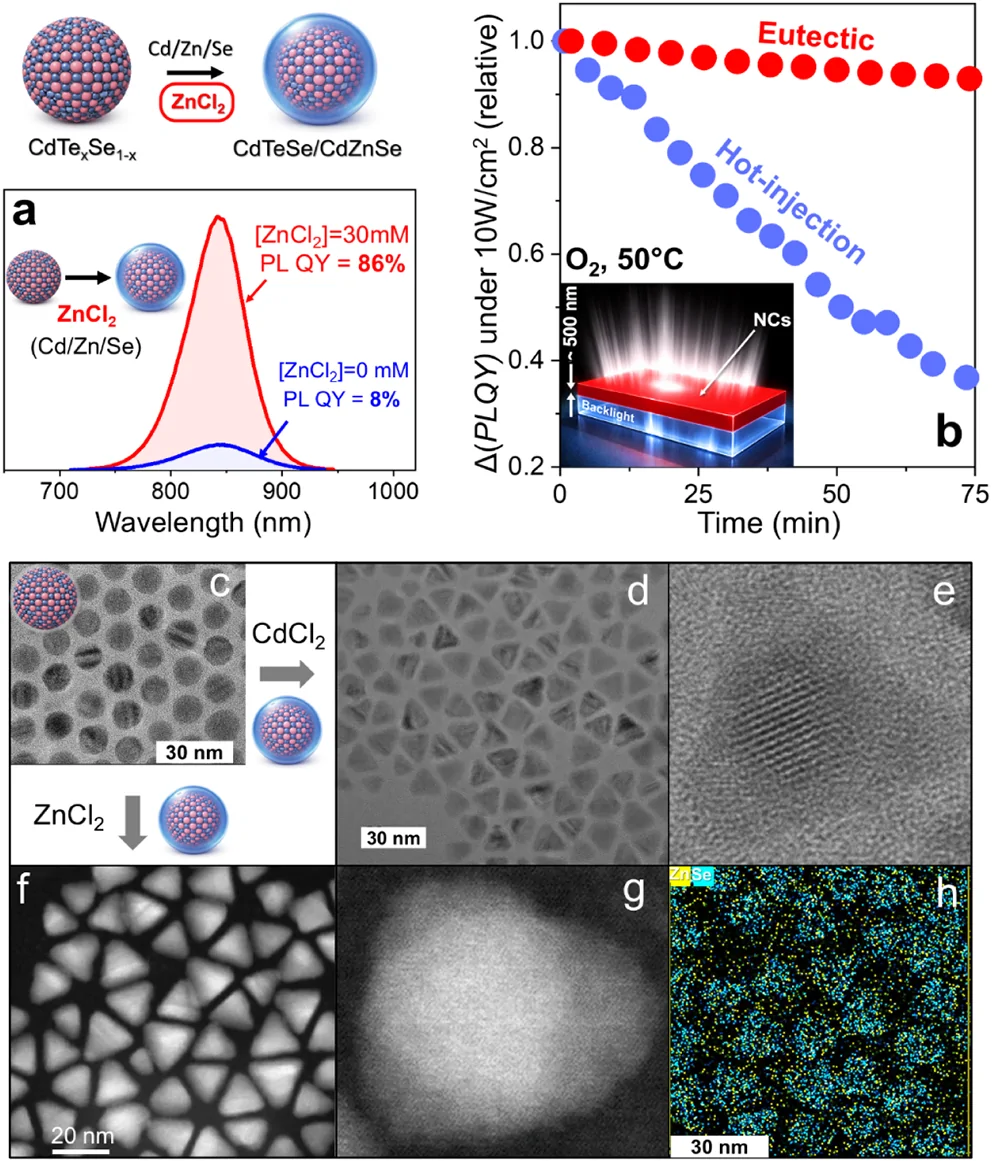
(a) Comparison of the PL intensity and QY between CdTe_0.78_Se_0.22_/Zn*_y_* Cd_1–*y*_Se core/shell QDs grown by a standard hot-injection method (blue) and those grown in the presence of ZnCl_2_ (red). The halide-assisted route increases PL QY from ∼8% to ∼86%. (b) Time evolution of PLQY for eutectic-processed (red) and untreated (blue) NC films (∼500 nm thick) under 50 °C and 10 W cm^–2^ laser irradiation. Films were unencapsulated (air exposure). (c) TEM images of CdTe*_x_* Se_1–*x*_ QDs. (d, e) TEM images of CdTe_0.78_Se_0.22_/Zn*_y_* Cd_1–*y*_Se core/shell NCs grown with CdCl_2_. (f, g) STEM characterization of CdTe_0.78_Se_0.22_/Zn*_y_* Cd_1–*y*_Se core/shell NCs grown with ZnCl_2_. (h) EDXS image depicting the elemental distribution of Zn and Se in the periphery of the CdTe_0.78_Se_0.22_/Zn*_y_* Cd_1–*y*_Se core/shell heterostructures. Adapted from ref. 58 available under a CC BY 4.0 license. Copyright 2026 The Authors.

Related halide-based growth methods have also been applied to other core/shell systems. For example, during the growth of InP/ZnSe core/shell NCs, the introduction of ZnF_2_ was shown to repair interfacial defects through halide ion penetration, improving the quality of the core/shell boundary.^105^ Eutectic processing has also been reported to enhance the photostability of CdSe/CdS core/shell NCs, an important requirement for display applications. After approximately 1 h of excitation-light exposure (Figure 6b), conventionally grown CdSe/CdS samples typically retain only ∼30% of their initial PL intensity, corresponding to a ∼ 70% loss (blue circles).^106^ By contrast, NCs growth in the presence of halide salts^58^ retained ∼ 96% of their initial PL (a ∼ 4% drop; Figure 6b, red circles), indicating that the eutectic route yields core/shell interfaces resilient to photothermal stress in an unencapsulated configuration.

Recently, eutectic processing of colloidal NCs has facilitated the development of a novel NC architecture known as a quantum shell (Figure 7).^66–68,107–115^ In quantum shells, the carrier confinement is realized within a thin, two-dimensional shell layer (e.g., CdSe) grown around a bulk-size, CdS core (Figure 7b). This geometry distributes multiexciton wave functions over a relatively large effective volume, thereby reducing carrier interactions and dramatically suppressing Auger recombination—an energy loss mechanism in colloidal NCs that has long limited their transition into optoelectronic technologies.,^116^

**Figure 7:**
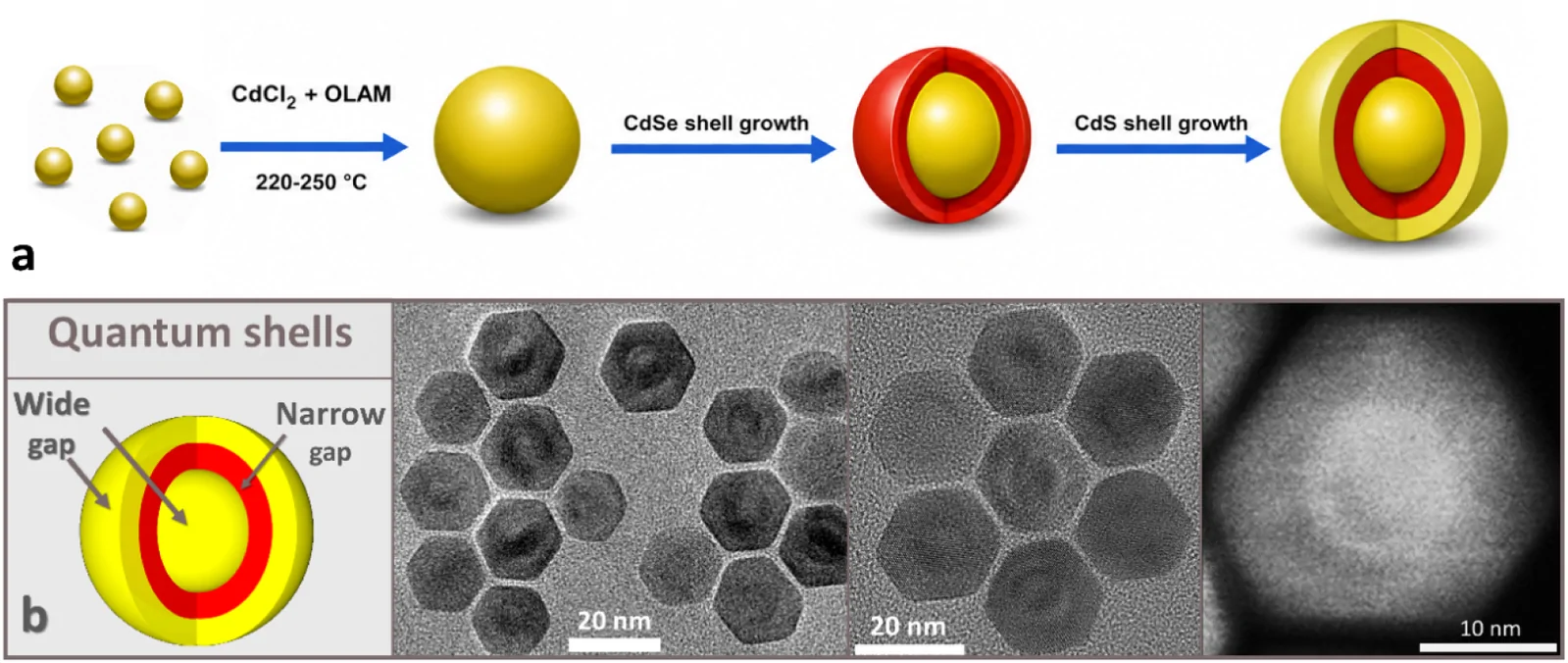
(a) Schematic illustration of quantum shell synthesis, showing the formation of a large single-crystalline CdS core through the coalescence of small CdS NCs in a CdCl_2_/OLAM mixture. (b) Structural representation of a semiconductor quantum shell, along with representative TEM images of CdS/CdSe/CdS quantum shells.

The key challenge associated with the synthesis of semiconductor quantum shells lies in the fabrication of a large, single-crystalline core that can serve as an epitaxial template for a quantum-confined shell. Conventional kinetically controlled syntheses often struggle to produce such large nanostructures with the required monocrystalline compositions. By contrast, eutectic-like growth proceeds through the coalescence of smaller NCs into larger semiconductor colloids (Figure 7a), followed by their thermodynamic reconstruction into relaxed morphologies with improved crystallinity. The size of the resulting core domain is controlled by only the reaction temperature. Meanwhile, the geometry of the core can be directed toward either a spherical or cubic^117^ shape by tuning the coordinating solvent. Subsequent epitaxial shell deposition on these high-quality templates yields distinct 2D quantum-confined layers (Figure 7b).

### Industrial Relevance of Eutectic NC Processing

Eutectic processing of colloidal NCs is beginning to impact commercial technologies that require improved photostability or a stronger Auger suppression. One representative example is the eutectic synthesis of CdTe_0.78_Se_0.22_/Zn_*y*_Cd_1–*y*_Se NCs, discussed above, for use as spectral-shaping material in the CdTe photovoltaics industry. Unlike the front side of commercial CdTe solar panels, which is designed for efficient light absorption, the backside, or film side exhibits external quantum efficiency (FS-QE) with a narrow maximum near 850 nm and a comparatively weak response across the visible spectral range (Figure 8b, blue curve). This spectral mismatch creates an opportunity for downconversion: a layer of NCs that absorbs visible photons and re-emits near 850 nm can redirect otherwise poorly harvested light into a wavelength range more efficiently collected by the device. To realize this function, eutectic-like growth conditions were used to synthesize ternary CdTe_0.78_Se_0.22_ alloy NCs with a high degree of homogeneous anion mixing, enabling the narrow PL line width required for spectral matching to the CdTe module response. As shown in Figure 8a, depositing CdTe_0.78_Se_0.22_/Zn_*y*_Cd_1–*y*_Se NCs on the film side of a solar panel broadens the FS-QE and produces pronounced visible range enhancement (Figure 8b, yellow curve). By converting high-energy photons into NIR, the NC layer increases the film-side short-circuit current from 3.3 to 9 mA cm^–2^ (Figure 8c). This gain corresponds to a 0.8–1.2% absolute gain in power conversion efficiency of CdTe photovoltaic modules.^58^

**Figure 8:**
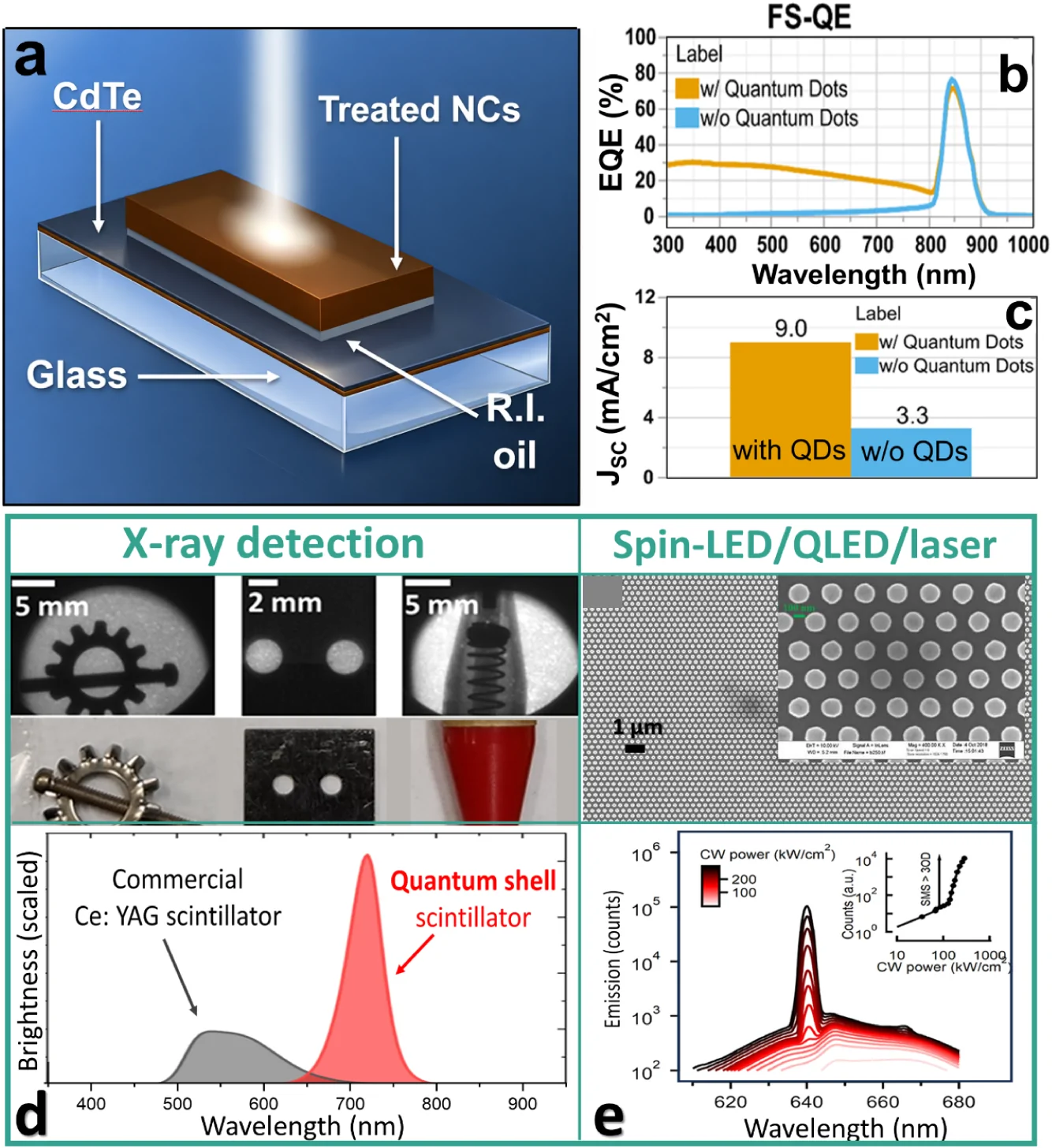
Representative optoelectronic applications of eutectic-processed NCs. (a-c) Solar energy downconversion for film-side CdTe PVs, including (a) schematic of the experimental setup for measuring the film-side external quantum efficiency (FS-EQE) of a CdTe solar cell modified with spectral downconversion NCs. (b) FS-QE of a commercial CdTe solar cell with and without CdSeTe QDs. (c) Film-side short-circuit current of the CdTe solar cell with and without QD downconverters. (d) X-ray detection using a quantum shell scintillator: upper panel, images of representative objects acquired using a tabletop X-ray tube source and camera; lower panel: radioluminescence of quantum shells compared with a Ce standard, normalized to the absorbed X-ray flux. (e) Emission spectra from quantum shells integrated into a photonic-crystal device with a 385 nm period under quasi-CW excitation using 7 ns, 532 nm pulses. The inset shows the increase in peak counts with pump fluence. Panels (a, b, c) adapted from ref. 58 available under a CC BY 4.0 license. Copyright 2026 The Authors. Panel (d) adapted from ref. 66 under the terms of the Creative Commons CC BY License. Panel (e) adapted from ref. 67 Copyright 2024 American Chemical Society.

Eutectic processing of NCs has also proven useful in X-ray scintillation detectors (Figure 8d). Solution-processed detectors of ionizing radiation including X-rays, γ rays, and β-particles, are becoming increasingly more attractive because of their relatively low cost and compatibility with large-area deposition.^118–120^ However, active materials processed from colloidal solutions are generally less stable under prolonged high-energy irradiation than conventional ceramic scintillators. In conventional NCs, lattice defects and surface traps can act as nonradiative recombination centers, reducing light yield and creating sites for radiation-induced degradation. Eutectic-processed NCs can address these limitations by combining improved crystallinity with reduced trap densities. As recently demonstrated by several groups,^66,121^ X-ray scintillators based on CdS/CdSe/CdS quantum shells containing thermodynamically grown CdS core domains provide brighter and significantly more durable scintillation for X-ray imaging technologies than many other colloidal nanomaterials.

Optical gain media based on colloidal NCs represent another important area where eutectic processing can offer significant advantages. Achieving optical gain requires high carrier densities, which, in turn, can magnify the detrimental effects of Auger recombination^122^ and photoinduced degradation.^123^ Consequently, the structural quality of the gain medium becomes a critical determinant of lasing performance. In this regard, eutectic synthesis can offer benefits by yielding NCs with relatively low lattice defect densities and geometries that are optimized for multiexciton processes. The potential of this approach has recently been demonstrated in two studies. In one example, Geiregat, Hens, and co-workers^59^ used eutectic-assisted coalescence of PbS NCs in solutions of lead halides and oleylamine to produce large, ∼20 nm PbS NCs featuring thermodynamically relaxed, low-energy structures that had been difficult to access using conventional synthetic methods.^124^ Ultrafast mid-infrared spectroscopy revealed room-temperature stimulated emission extending to the bulk PbS band edge near 2.95 μm, with net gain approaching 1000 cm^–1^ and a gain lifetime of approximately 0.5 ns. In a related study, the same group demonstrated that CdS/CdSe/CdS quantum shells incorporating eutectic-processed core NCs provide a robust optical gain medium capable of operating efficiently in the high excitation density multiexciton regime.^67^ Their low-defect architecture, together with suppressed Auger recombination, enabled the demonstration of an elusive quasi-CW stimulated emission.

### A Phenomenological Mechanism of NC-Halide Eutectics

Although the molecular details of eutectic processes in NCs depend on the nanoparticle geometry and composition, their overall behavior can be described within a common phenomenological framework derived from bulk semiconductor systems.^125,126^ As illustrated in Figure 9a, the interaction of NCs with halide salts can be qualitatively represented using the eutectic phase diagram,^127^ where the horizontal axis denotes the halide-to-semiconductor NC ratio and the vertical axis represents the reaction temperature. The exact phase boundaries are expected to depend on NC size, crystal structure, and ligand environment and may also be influenced by halide-enhanced ion mobility at nanoscale interfaces. Despite these system-specific effects, the diagram can be divided into four qualitative regimes that capture the principal stages of the eutectic-assisted nanocrystal transformation. At temperatures below the eutectic threshold, *T* < T_eut_, NCs and halide additives remain in their solid states regardless of the halide/NC ratio. Upon heating into the intermediate temperature range, T_eut_ < *T* < T_NC_, low halide concentrations can produce a regime in which the halide component becomes mobile or liquid-like while the semiconductor NC lattice remains crystalline. In this regime, partially solvated halides can wet the NC surface and promote the migration of both cations and anions along the particle interface. Such a state is consistent with the observed improvement in PL characteristics of core/shell NCs processed at moderate temperatures.^58^ Increasing the halide content shifts the system toward lower effective NC melting temperatures, as indicated in Figure 9a. This shift lowers the onset temperature for NC coalescence, T_eut_, consistent with approaching the eutectic-like regime. The same trend is reflected experimentally in Figure 3e, where increasing CdCl_2_ concentration progressively reduces CdSe coalescence threshold before the effect saturates. At even higher temperatures, both the halide-rich phase and the semiconductor NC phase become liquid-like, enabling extensive intermixing, full particle liquefaction, and subsequent recrystallization into thermodynamically favored structures.

**Figure 9:**
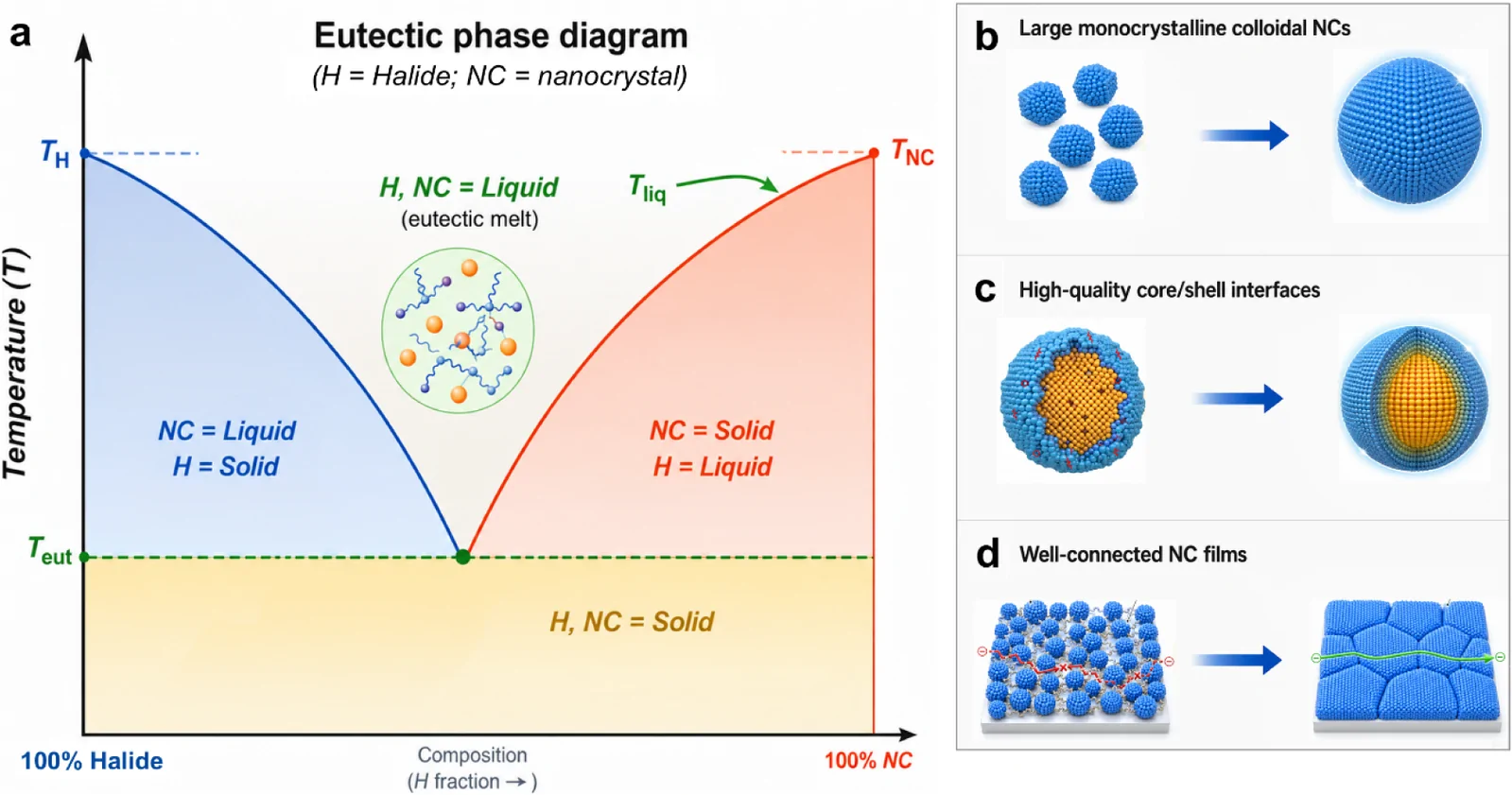
(a) Qualitative eutectic phase diagram for halide (H)–nanocrystal (NC) mixtures, showing the halide fraction on the *x*-axis and reaction temperature on the *y*-axis. (b-d) Potential applications of eutectic processing in colloidal NC synthesis. (b) Eutectic-assisted coalescence of small NCs into large, single-crystalline nanocrystals with reduced defect densities and thermodynamically relaxed morphologies. (c) Local eutectic reconstruction at strained core/shell interfaces can promote interfacial reorganization, yielding high-quality heterostructured NCs. (d) Eutectic processing of NC assemblies can offer a potential route toward epitaxially connected NC films, in which initially ligand-separated particles are transformed into grain-like networks with improved charge transport pathways. Panel (a) adapted from ref. 58 available under a CC BY 4.0 license. Copyright 2026 The Authors.

## Outlook

Eutectic-assisted synthesis has emerged as a powerful strategy in colloidal NC chemistry. Its principal advantage lies in the ability to lower the energetic barriers for lattice reorganization, thus allowing NCs to escape kinetically trapped configurations and recrystallize into lower defect, thermodynamic structures. To date, the best demonstrated benefits of this approach include the formation of high-quality binary and ternary NCs through coalescence and recrystallization (Figure 9b) and the reduction of interfacial strain in core/shell heterostructures (Figure 9c).

In the near term, the development in this emerging area is likely to focus on identifying appropriate eutectic mixtures for specific semiconductor lattices. These advances should enable expansion into more challenging compositions, such as III–V NCs, I–III–VI, and complex multinary alloys. Halide-improved crystallization of grain boundaries was also recently reported in developing perovskite solar cells.^128^ Equally important will be determining the temperature, coordinating ligand environment, and other reaction conditions that promote reconstruction without decomposition. For instance, the influence of nanoscale interfaces on halide—lattice interactions and the underlying thermodynamics remains poorly understood. An important challenge is therefore to distinguish halide-induced bond breaking and enhanced lattice mobility from true melting or other phase transitions. Developing in situ probes capable of directly identifying transient liquid-like states will be essential for establishing a more complete mechanistic picture of these transformations.

A particularly exciting direction is the eutectic transformation of solid NC films. In principle, eutectic processing could convert ligand-separated nanocrystal assemblies into electronically coupled, epitaxially connected solids, analogous in concept to the recrystallization strategies used in CdTe photovoltaics. Such an approach would enable the solution processing of colloidal nanomaterials while introducing the improved electronic transport associated with polycrystalline semiconductors.^129^

Ultimately, employing eutectic mixtures during NC synthesis will challenge the conventional view of colloidal NCs as products of irreversible kinetic growth. Instead, it suggests that NCs can be chemically reshaped after synthesis through carefully designed liquid-like transformations. This concept opens a broad material space for producing high-performance NCs and their solids that are difficult, or perhaps impossible, to achieve using conventional colloidal methods.

## Conclusions

Eutectic reactions offer a new framework for the synthesis and transformation of colloidal semiconductor NCs. By promoting halide-based lattice softening and the subsequent recrystallization, these processes can remove structural defects or induce thermodynamic phase transformations in NCs. The method has already produced high-quality Cd, Pb, and Zn chalcogenide NCs as well as alloyed and heterostructured materials with promising performance in light-emitting and energy-related technologies. At the same time, the field is still at an early stage and its mechanistic foundations remain under active development. Initial studies nevertheless suggest that eutectic processing can overcome several long-standing limitations of the conventional NC synthesis. Its impact could become particularly significant if the approach is extended to III–V compositions and to the formation of epitaxially connected NC solids. In this broader view, eutectic processing is not merely a postsynthetic treatment but a general materials chemistry strategy for converting kinetically formed colloidal NCs into thermodynamically refined materials.
